# The Association of Lipoprotein(a) with Major Adverse Cardiovascular Events after Acute Myocardial Infarction: A Meta-Analysis of Cohort Studies

**DOI:** 10.31083/RCM27376

**Published:** 2025-05-15

**Authors:** Huiruo Liu, Liangshan Wang, Hong Wang, Xing Hao, Zhongtao Du, Chenglong Li, Xiaotong Hou

**Affiliations:** ^1^Centre for Cardiac Intensive Care, Beijing Institute of Heart, Lung and Blood Vessel Diseases, Beijing Anzhen Hospital, Capital Medical University, 100029 Beijing, China

**Keywords:** lipoprotein(a), acute myocardial infarction, major adverse cardiovascular events, meta-analysis

## Abstract

**Background:**

Despite evidence suggesting a link between lipoprotein(a) (Lp(a)) and the occurrence of acute myocardial infarction (AMI), the relationship regarding prognoses related to AMI remains unclear. This meta-analysis was conducted to summarize the association between Lp(a) and the risks of major adverse cardiovascular events (MACEs) among populations surviving AMI.

**Methods:**

We searched PubMed, Embase, Web of Science, MEDLINE, and Cochrane Library databases until February 14, 2024. Cohort studies reporting multivariate-adjusted hazard ratios (HRs) for the correlation of Lp(a) with MACEs in AMI populations were identified. The Lp(a) level was analyzed using categorical and continuous variables. Subgroup analyses were conducted based on gender, type of AMI, diabetic and hypertensive status. Publication bias was assessed using funnel plots. A random-effect model was utilized to pool the results.

**Results:**

In total, 23 cohorts comprising 30,027 individuals were recruited. In comparison to those categorized with the lowest serum Lp(a), individuals in the highest category showed higher risks of MACEs after AMI (HR: 1.05, 95% confidence interval (CI): 1.01–1.09, *p* = 0.006). Similar findings were exhibited when Lp(a) was analyzed as a continuous variable (HR: 1.14, 95% CI: 1.02–1.26, *p* = 0.02). Subgroup analyses indicated that this correlation persisted significantly among females (HR: 1.23, *p* = 0.005), diabetes mellitus (DM) (HR: 1.39, *p* = 0.01), hypertension (HR: 1.36, *p* < 0.00001), ST-segment elevation myocardial infarction (STEMI) (HR: 1.03, *p* = 0.04), non-STEMI (HR: 1.40, *p* = 0.03), and long-term (>1 year) MACE (HR: 1.41, *p* = 0.0006) subgroups.

**Conclusions:**

Higher Lp(a) levels might be an independent indicator for MACE risks after AMI, especially among female populations with DM and/or hypertension, and more suitable for evaluating long-term MACEs.

**The PROSPERO Registration:**

CRD42024511985, https://www.crd.york.ac.uk/prospero/display_record.php?ID=CRD42024511985.

## 1. Introduction

Even after undergoing optimal medical treatment in line with the latest guidelines, individuals who survive an acute myocardial infarction (AMI) continue to face a less-than-favorable prognosis globally [1, 2]. Consequently, identifying valid residual risk indicators for major adverse cardiac events (MACEs) following AMI is paramount [3]. Indeed, poorly controlled dyslipidemia has been highlighted among the primary drivers of subsequent MACEs since it is significantly influenced by the accumulation of not only low-density lipoprotein (LDL) cholesterol but also other cholesterol-rich apolipoproteins within the vessel wall, such as lipoprotein(a) (Lp(a)) [4, 5].

Lp(a) possesses a core composition similar to that of LDL, containing approximately 30% to 46% cholesterol and oxidized phospholipids, which promotes cholesterol deposition in coronary walls, triggers inflammatory responses, and enhances thrombogenic potential [6]. Recent studies have confirmed that elevated Lp(a) levels are associated with increased risks and adverse coronary artery disease outcomes [7, 8], which remains readily comprehensible for the established correlations of Lp(a) with in-stent restenosis [9], accelerated progression of atherosclerotic plaques, and more severe coronary calcification [10, 11]. Moreover, higher Lp(a) levels have also been linked to other various cardiovascular disorders, such as aortic valve calcification [12] and left ventricular hypertrophy [13]. Notably, as demonstrated in a previous meta-analysis, elevated Lp(a) levels were associated with an increased prevalence of MACEs among individuals with coronary heart disease [14] and higher rates of all-cause and cardiac mortality in the general population [15]. More importantly, Bittner *et al*. [16] found that reductions in the baseline Lp(a) level could predict the risk of MACEs after recent acute coronary syndrome. However, despite these findings, the clinical significance of Lp(a) in the prognosis of AMI remains controversial. Relevant studies have reported inconsistent results [17, 18, 19, 20, 21, 22, 23, 24, 25, 26, 27, 28, 29, 30, 31, 32, 33, 34, 35, 36, 37, 38, 39]; some concluded that elevated Lp(a) levels are associated with an increased risk of MACEs following AMI, whereas others found no significant association between the two.

Given the potential of Lp(a) as a modifiable risk factor, this meta-analysis aimed to synthesize existing cohort studies concerning the relationship between baseline Lp(a) levels and the subsequent risk of MACEs in the AMI population. The objective was to provide a comprehensive understanding of the role of serum Lp(a) levels in AMI prognosis, evaluate its potential as a prognostic marker, and lay the foundation for further validation regarding any underlying mechanisms.

## 2. Materials and Methods

The current review was reported per the PRISMA guidelines [40] (PROSPERO: CRD42024511985).

### 2.1 Literature Search

A systematic search was performed in PubMed, Embase, Web of Science, MEDLINE, and Cochrane Library databases from inception to February 14, 2024, without language restriction, using the combination of search terms: (1) “Lipoprotein(a)” OR “Lipoprotein Lp(a)” OR “Lipoprotein (a)” OR “Lipoprotein a” OR “Lp(a)” and (2) “heart infarction” OR “myocardial infarction” OR “myocardial infarct” OR “cardiovascular stroke” OR “heart attack” OR “MINOCA” OR “cardiogenic shock”. Manual hand-searching of gray literature and reference lists from relevant studies was complemented. An elaborate search strategy is presented in **Supplementary Table 1**.

### 2.2 Selection Criteria

Two reviewers conducted an independent screening of the literature. Disagreements between the reviewers were resolved through discussion, and a third reviewer was consulted if necessary. Studies satisfying the below criteria were incorporated: (P) population: adult individuals with AMI at admission; (E) exposure and (C) comparator: high (higher than cut-off values of 10.3 mg/dL or the highest tertile ranging from 28.7 to 134.4 mg/dL) versus low serum Lp(a) level; (O) outcomes: MACEs, cardiovascular death, recurrent myocardial infarction (MI), or all-cause death. A MACE was defined as a composite of all-cause death, nonfatal MI, nonfatal stroke, hospitalization for heart failure (HF), and revascularization. Exclusion criteria included: (1) cross-sectional research or conference abstracts; (2) studies not reporting multi-adjusted hazard ratios (HRs) for the correlation of serum Lp(a) with the above-mentioned outcomes; (3) studies not in English. In instances where there was an overlap in the populations of different studies derived from the same registry or group, only the sample with the largest size was included.

### 2.3 Data Extractions and Quality Assessments

Two investigators independently extracted and cross-checked data from retrieved articles, with any discrepancies resolved through discussion or reference by a third investigator. Data extracted were as follows: (1) first author’s name, publication year, study design, stratification criteria for Lp(a) levels, and follow-up duration; (2) patient characteristics, including research region, sample size, age, sex, AMI subtype, and revascularization rate; (3) patterns of serum Lp(a) analysis, confounders adjusted, and outcomes reported. The quality (article selection (a maximum of 4 points, with 2 points for the representativeness of the exposed cohort and 2 points for the selection of the non-exposed cohort and the ascertainment of exposure), comparability (a maximum of 2 points, with 1 point for the adjustment of age and 1 point for the adjustment of other confounding factors), and outcomes (a maximum of 3 points, with 2 points for the assessment of outcomes and 1 point for the duration of follow-up)) of enrolled cohorts were evaluated via the Newcastle–Ottawa scale (NOS) [41]. No minimum inclusion score threshold was set for the NOS.

### 2.4 Statistics Analysis

HRs and 95% confidence intervals (CIs) were used to indicate the relationship between serum Lp(a) and outcomes of individuals with AMI. For studies analyzing Lp(a) as categorical variables, HRs comparing the occurrence of outcomes in populations in the highest Lp(a) category to those in the lowest Lp(a) category were collected. For studies analyzing Lp(a) as continuous variables, HRs for incidences of outcomes per 1-unit increment in Lp(a) level were collected. The HRs were logarithmically transformed, and the standard errors were derived from the 95% CIs. Cochran’s Q test and I^2^ statistics assessed the heterogeneity, with statistical significance considered when I^2^ > 50 or *p* < 0.10. Subgroup and sensitivity analyses were conducted to control potential heterogeneity further, and a random-effects model was employed to synthesize the HR data. Subgroup analyses were conducted based on population characteristics, such as sex, type of AMI, and diabetic and hypertensive status, as well as the association between serum Lp(a) level and MACE risks. Publication bias was assessed visually using funnel plots. RevMan (Version 5.4, The Cochrane Collaboration, Copenhagen, Denmark) software was used to carry out these analyses.

## 3. Results

### 3.1 Literature Search

**Fig. 1.**
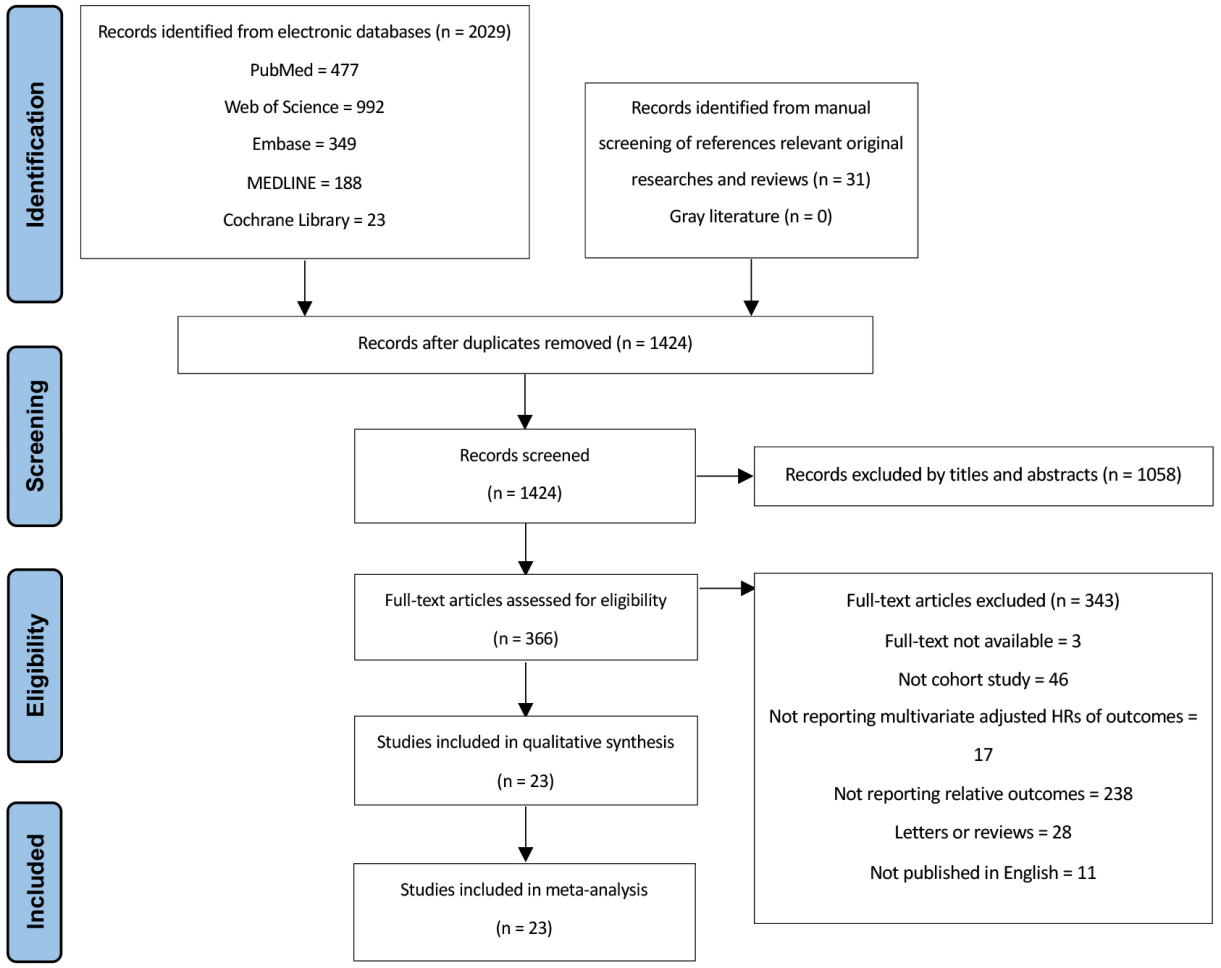
**PRISMA flowchart for study selection**. HRs, hazard ratios.

Fig. 1 illustrates the progress of conducting a comprehensive literature search. Initially, a sum of 2060 articles were searched. After removing duplicate articles, 366 papers were thoroughly examined in full-text format. Of those, 23 cohorts were recruited for subsequent analyses [17, 18, 19, 20, 21, 22, 23, 24, 25, 26, 27, 28, 29, 30, 31, 32, 33, 34, 35, 36, 37, 38, 39].

### 3.2 Study Characteristics and Quality Assessment

The characteristics of cohorts enrolled in our analysis are presented in Table 1 (Ref. [17, 18, 19, 20, 21, 22, 23, 24, 25, 26, 27, 28, 29, 30, 31, 32, 33, 34, 35, 36, 37, 38, 39]). In total, 23 cohorts (14 prospective and nine retrospective) were recruited in the current review, comprising 30,027 individuals diagnosed with acute myocardial infarction (AMI) upon admission. Studies included in the meta-analysis were published between 1998 and 2023; 17 were performed in Asian countries, while another six were carried out in Europe. Across the 23 trials, sample sizes varied from 66 to 8295, and median age varied between 55.7 and 67.7 years, with the proportions of males ranging from 70.5% to 83.9%, ST-segment elevation myocardial infarction (STEMI) diagnosis ranging from 36.4% to 100.0%, and the population receiving revascularization ranging from 27.0% to 100.0%. The median follow-up varied from the length of stay in the hospital to 88 months. Baseline serum Lp(a) was calculated as categorical variables in 17 cohorts [17, 18, 19, 20, 21, 22, 23, 24, 26, 28, 30, 31, 32, 35, 36, 37, 38], as a continuous variable in only one cohort [34], and as both in five cohorts [25, 27, 29, 33, 39]. HRs for the association between prognosis and Lp(a) were adjusted utilizing age, sex, body mass index, blood pressure, medical history, laboratory and angiographic findings, and in-hospital medications to varying degrees. The NOS score for all cohorts enrolled was 9, demonstrating high levels of quality (Table 2, Ref. [17, 18, 19, 20, 21, 22, 23, 24, 25, 26, 27, 28, 29, 30, 31, 32, 33, 34, 35, 36, 37, 38, 39]).

**Table 1.** **Baseline characteristics of included studies**.

Study, publication year	Design	Follow-up duration (months)	Number of participants	Data source	Median age (years)	Male (%)	STEMI (%)	Revascularization (%)	Serum Lp(a) analysis and stratification criteria for Lp(a)	Outcomes	Variables adjusted
Stubbs, 1998 [17]	PC	36.0	266	London (1995–1998)	63.0	77.0	100	27.0	Median (30 mg/dL)	Cardiac mortality	Age, prior MI, infarct size, HP, thrombolysis, revascularization, beta-blocker, aspirin, intravenous heparin on CCU
Igarashi, 2003 [18]	PC	35.0	127	Japan (1996–2001)	61.0	81.9	100	100.0	Median (47 mg/dL)	MACEs	Age, sex, DM, HP, smoking, hypercholesterolemia drinking, number of diseased vessels, Killip class, reperfusion time, door to balloon time, LVEF
Gómez, 2009 [19]	PC	6.0	1271	Spanish (N/A)	57.1	83.9	77.1	71.8	T3:T1 (48 mg/dL)	MACEs	Age, sex, hypercholesterolemia, DM, HP, oxidized-LDL, smoking
Cho, 2010 [20]	RC	12.0	832	Korea (2005–2007)	62.8	72.1	N/A	100.0	T3:T1 (31 mg/dL)	MACEs	Age, sex, smoking, TC, LDL, Apo B
Ikenaga, 2011 [21]	RC	60.0	410	Japan (1999–2007)	63.2	82.4	100.0	100.0	Median (40 mg/dL)	MACEs, ReMI	Age, sex, DM, HP, smoking, prior MI, Killip class >1, time to angiography, anterior MI, initial TIMI 0–1flow, final TIMI 3, CKD, MVD
Peng, 2017 [22]	PC	12.0	175	China (N/A)	59.6	82.9	100.0	100.0	Median (30 mg/dL)	MACEs	Age, sex, smoking, SBP, DBP, TC, Cr
Mitsuda, 2019 [23]	RC	36.0	668	Japan (2007–2014)	65.8	80.5	100.0	100.0	Median (50 mg/dL)	MACEs	Age, sex eGFR, CRP, prior CAD
Sumarjaya, 2020 [24]	PC	Length of hospital stay	66	Indonesia (2018)	59.2	80.3	63.6	48.4	Median (10.3 mg/dL)	MACEs	Age, sex, HP, DM, dyslipidemia, smoking, obesity, CKD, reperfusion therapy
Cao, 2021 [25]	PC	50.4	3864	China (2009–2019)	61.7	79.7	58.6	74.9	Q4:Q1 (41 mg/dL) continuous	MACEs, cardiac mortality	Age, sex, BMI, family history of CAD, HP, smoking, DM, pre-revascularization, Gensini score, LDL-C, TG, FBG, hs-CRP, baseline statin use
Galasso, 2021 [26]	PC	66.9	724	Italy (2014–2019)	62.1	77.3	100.0	100.0	Median (50 mg/dL)	ReMI	Age, sex, DM, history of CAD, multivessel disease, restenosis lesion
Gao, 2021 [27]	PC	41.7	1179	China (2015–2019)	55.7	74.0	53.0	73.5	T3:T1 (30 mg/dL)	MACEs	Age, sex, AMI type, HP, DM, dyslipidemia
									continuous		
Liu, 2021 [28]	RC	60.0	8295	China (2007–2018)	N/A	N/A	N/A	N/A	Median (15 mg/dL)	All-cause mortality	Age, sex, HP, prior MI, DM, prior PCI, Hb, WBC, CHF, TC, TG, Apo A, LDL-C, HDL-C, CKD, angiotensin-converting enzyme inhibitor/angiotensin receptor blockers, β-blockers, statins
Wang, 2021 [29]	RC	30.0	2318	China (2012–2017)	58.8	79.8	100.0	100.0	T3:T1 (T3 ranging from 28.7 to 134.4 mg/dL)	MACEs	Age, sex, HP, DM, TC, TG, LDL-C, HDL-C
									continuous		
Wohlfahrt, 2021 [30]	PC	19.0	851	Czech (2017–2020)	65.1	75.4	57.8	92.1	T3:T1 (56.3 mg/dL)	All-cause mortality	Age, sex, BMI, smoking, LDL-C, HP, DM, Cr, Killip class
Xue, 2021 [31]	PC	31.0	1359	China (2015–2018)	63.4	79.5	100.0	100.0	T3:T1 (19.1 mg/dL)	All-cause mortality	Age, sex, HP, dyslipidemia, smoking, DM, CKD, symptom onset to balloon, BMI, SBP, HbA1c, TG, TC, HDL-C, LDL-C, CK-MB, Cr, hs-CRP, LVEF, prehospital thrombolysis, lipid-lowering medication
Yoon, 2021 [32]	PC	88.8	1650	Korea (2003–2013)	N/A	N/A	N/A	100.0	Median (30 mg/dL)	MACEs	Age, sex, initial presentation, BMI, HP, DM, smoking, prior MI, prior stroke, prior PAD, CKD, baseline LVEF, left main disease, multivessel disease, enrollment period (year), LDL, HDL-C, antithrombotic and statin prescription at discharge
Silverio, 2022 [33]	PC	37.4	1018	Italy (2013–2019)	63.0	75.7	75.7	100.0	Q5:Q1 (70 mg/dL) continuous	All-cause mortality, ReMI	Age, sex, HP, hyperlipidemia, smoking, DM, history of CAD, obesity, AMI type, GFR at admission, TC, HDL-C, LDL-C, multivessel disease, treated vessel by PCI/CABG
Wang, 2022 [34]	RC	60.0	171	China (2014–2017)	N/A	N/A	N/A	100.0	continuous	MACEs	LVEF and eGFR
Dai, 2023 [35]	RC	55.2	262	Japan (2015–2018)	67.7	74.8	100.0	100.0	Median (32 mg/dL)	MACEs	Age, prior MI, Killip 2-4, TG, HbA1c, NT-proBNP, Hb, ACEI/ARB use, loop diuretic use, MRA use, LVEF
Park, 2023 [38]	PC	36.5	1908	Korea (2011–2015)	65.2	70.5	36.4	87.7	T3:T1 (50 mg/dL)	MACEs	Age, sex, BMI, HP, DM, smoking, family history of CAD, prior MI, prior HF, prior CVD, SBP, LVEF, Killip class >2, anemia, TC, LDL-C, eGFR, PCI, high-intensity statin
Rigattieri, 2023 [36]	RC	36.0	634	Italy (2018–2020)	N/A	N/A	N/A	100.0	Median (30 mg/dL)	MACEs	PAD, number of diseased coronary vessels, coronary chronic total occlusion
Zhang, 2023 [37]	RC	48.0	436	China (2018–2020)	65.0	71.4	49.5	100.0	T3:T1 (T3 ranging from 34.2 to 120 mg/dL)	All-cause mortality	Age, sex, hospitalization time, heart rate, DM, prior PCI, Hb, Apo-A, eGFR, uric acid, FBG
Li, 2023 [39]	PC	48.2	1543	China (2017–2020)	61.8	80.9	100.0	100.0	Median (30 mg/dL) continuous	MACEs, cardiac mortality, all-cause mortality, ReMI	Age, sex, BMI, HP, dyslipidemia, PAD, CKD, prior MI or PCI, Killip class, GRACE score, multivessel disease, eGFR, LVEF, TC, LDL-C, hs-CRP, cTnI, NT-proBNP

AMI, acute myocardial infarction; STEMI, ST-segment elevation myocardial infarction; ReMI, recurrent myocardial infarction; DM, diabetes mellitus; Lp(a), lipoprotein(a); RC, retrospective cohort; PC, prospective cohort; BMI, body mass index; MACEs, major adverse cardiovascular events; SBP, systolic blood pressure; DBP, diastolic blood pressure; WBC, white blood cells; eGFR, estimated glomerular filtration rate; LVEF, left ventricular ejection fraction; HDL-C, high-density lipoprotein cholesterol; HbA1c, glycosylated hemoglobin; PAD, peripheral vascular disease; CAD, coronary artery disease; CKD, chronic kidney disease; PCI, percutaneous coronary intervention; CABG, coronary artery bypass grafting; MRA, magnetic resonance angiography; TC, total cholesterol; TG, triglyceride; HP, hypertension; LDL-C, low-density lipoprotein cholesterol; NT-proBNP, N-terminal pro-brain natriuretic peptide; cTnI, cardiac troponin I; FBG, fasting blood glucose; hs-CRP, high-sensitivity C-reactive protein; MI, myocardial infarction; Cr, creatinine; Apo A, apolipoprotein A; Apo B, apolipoprotein B; CCU, cardiac care unit; MVD, microvascular disease; TIMI, thrombolysis and thrombin inhibition in myocardial infarction; HF, heart failure; CHF, chronic heart failure; N/A, not applicable; CK-MB, creatine kinase-MB; ACEI/ARB, angiotensin-converting enzyme inhibitor/angiotensin receptor blocker.

**Table 2.** **Newcastle–Ottawa Scale appraisal of included studies**.

Study, publication year	Selection	Comparability	Outcome	Total
Stubbs, 1998 [17]	4	2	3	9
Igarashi, 2003 [18]	4	2	3	9
Gómez, 2009 [19]	4	2	3	9
Cho, 2010 [20]	4	2	3	9
Ikenaga, 2011 [21]	4	2	3	9
Peng, 2017 [22]	4	2	3	9
Mitsuda, 2019 [23]	4	2	3	9
Sumarjaya, 2020 [24]	4	2	3	9
Cao, 2021 [25]	4	2	3	9
Galasso, 2021 [26]	4	2	3	9
Gao, 2021 [27]	4	2	3	9
Liu, 2021 [28]	4	2	3	9
Wang, 2021 [29]	4	2	3	9
Wohlfahrt, 2021 [30]	4	2	3	9
Xue, 2021 [31]	4	2	3	9
Yoon, 2021 [32]	4	2	3	9
Silverio, 2022 [33]	4	2	3	9
Wang, 2022 [34]	4	2	3	9
Dai, 2023 [35]	4	2	3	9
Park, 2023 [38]	4	2	3	9
Rigattieri, 2023 [36]	4	2	3	9
Zhang, 2023 [37]	4	2	3	9
Li, 2023 [39]	4	2	3	9

### 3.3 Serum Lp(a) and the Occurrence of MACEs

**Fig. 2.**
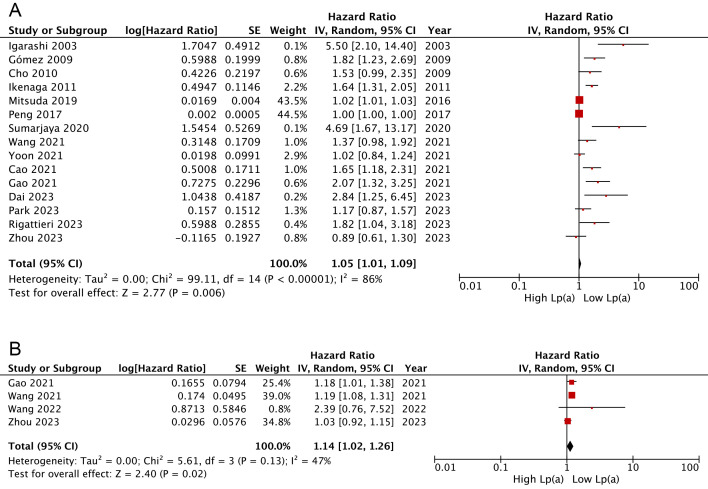
**Forest plots regarding meta-analyses of the links of Lp(a) with risks of composite MACEs**. (A) Meta-analyses with Lp(a) pooled as categorical variables. (B) Meta-analyses with Lp(a) pooled as continuous variables. IV, inverse variance; CI, confidence interval.

Overall, the combined findings of 15 cohorts [18, 19, 20, 21, 22, 23, 24, 25, 27, 29, 32, 35, 36, 38, 39] indicated that AMI patients categorized with the lowest serum Lp(a) presented a lower likelihood of experiencing MACEs, in comparison to those in the highest category (HR: 1.05, 95% CI: 1.01–1.09, I^2^ = 86%, *p* = 0.006) (Fig. 2A). Similar findings were observed when analyzing Lp(a) as a continuous variable (HR: 1.14, 95% CI: 1.02–1.26, I^2^ = 47%, *p* = 0.02; Fig. 2B) [27, 29, 34, 39].

**Fig. 3.**
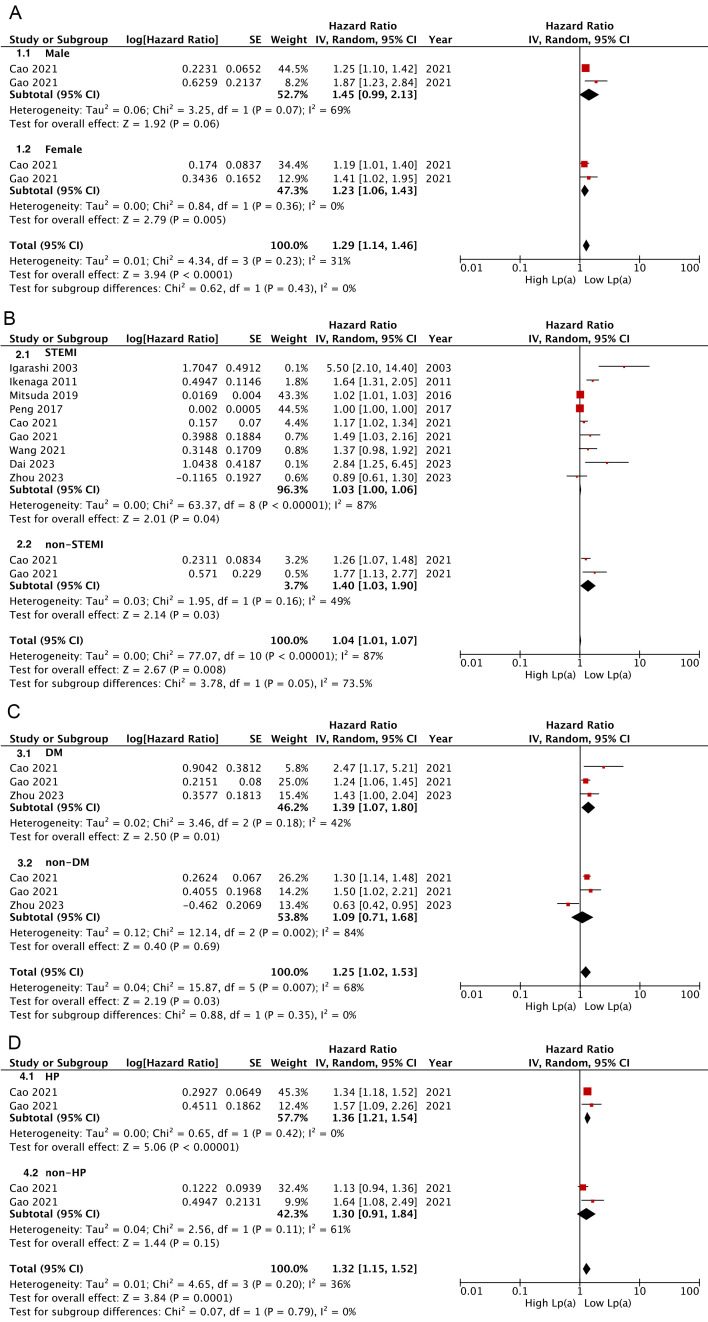
**Subgroup analysis for Lp(a) links analyzed as categorical variables with risks of composite MACEs**. (A) Subgroup analysis according to sex (A), AMI type (B), diabetes (C), and hypertension (D).

Subgroup analyses revealed that AMI patients with a higher Lp(a) category presented significantly increased risks of MACEs, regardless of the AMI subtype (STEMI: HR: 1.03, 95% CI: 1.00–1.06, *p* = 0.04; non-STEMI: HR: 1.40, 95% CI: 1.03–1.90, *p* = 0.03) (Fig. 3B); however, this elevated risk remained apparent only among female individuals and those with DM or hypertension (Fig. 3A,C,D). Further, pooled findings indicated a significant correlation between Lp(a) and long-term (>1 year) MACEs (HR: 1.41, 95% CI: 1.16–1.72, I^2^ = 84%, *p* = 0.0006), but not short-term (≤1 year) MACEs (HR: 1.62, *p *= 0.05) (**Supplementary Fig. 1**). Sensitivity analysis indicated that these associations remained relatively robust (**Supplementary Fig. 2**). In the subgroup analysis where Lp(a) was calculated as continuous variables, only studies with stratified populations based on the AMI type or diabetic status could be pooled for analysis; meanwhile the exhibited results aligned with those for when Lp(a) was calculated as a categorial variable (**Supplementary Fig. 3**).

### 3.4 Serum Lp(a) and the Occurrence of All-Cause Death, Cardiac Death, and ReMI

**Fig. 4.**
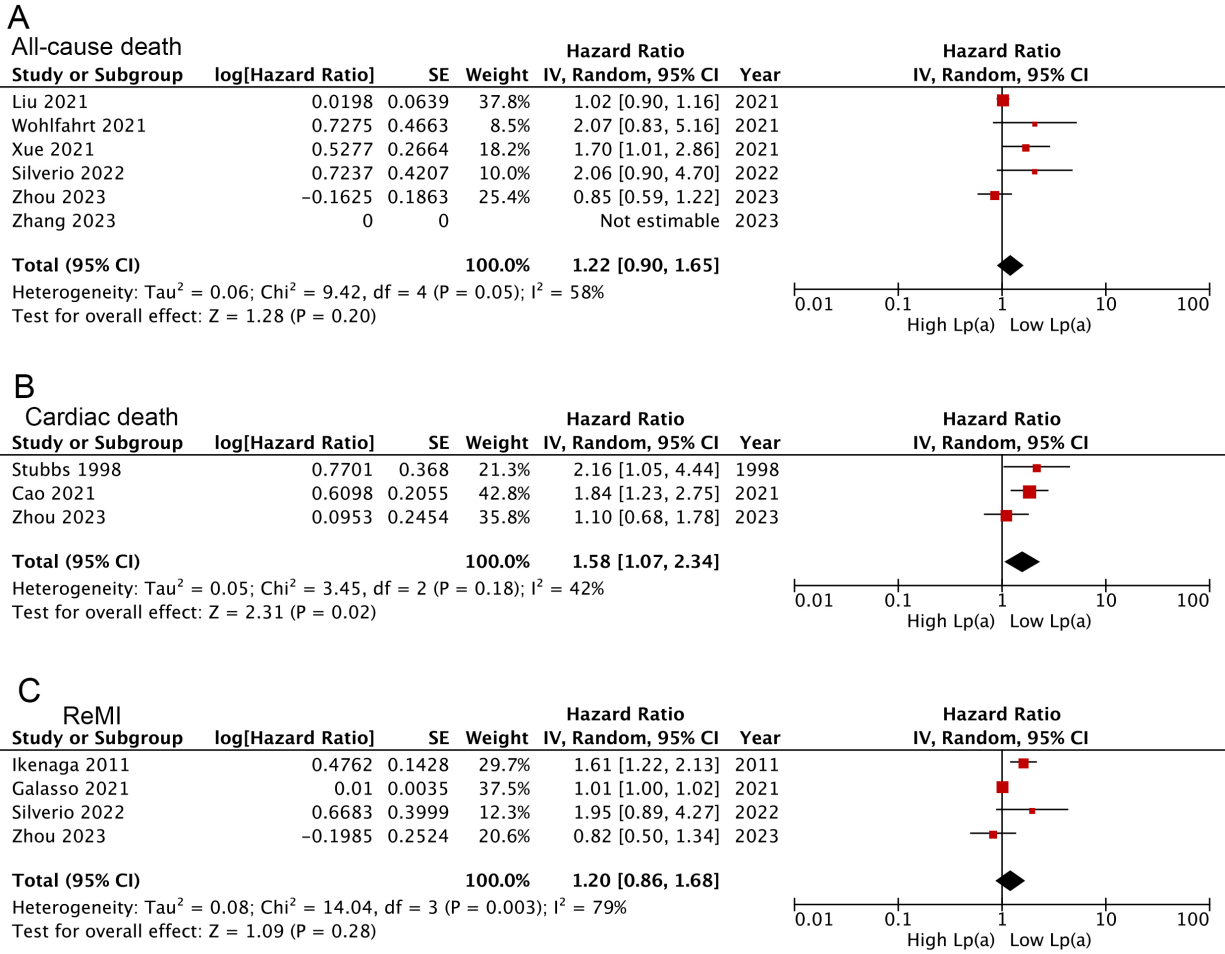
**Forest plots regarding meta-analyses for the links of Lp(a) analyzed as a categorical variable with the risk of all-cause death (A), cardiac death (B), and ReMI (C)**.

**Fig. 5.**
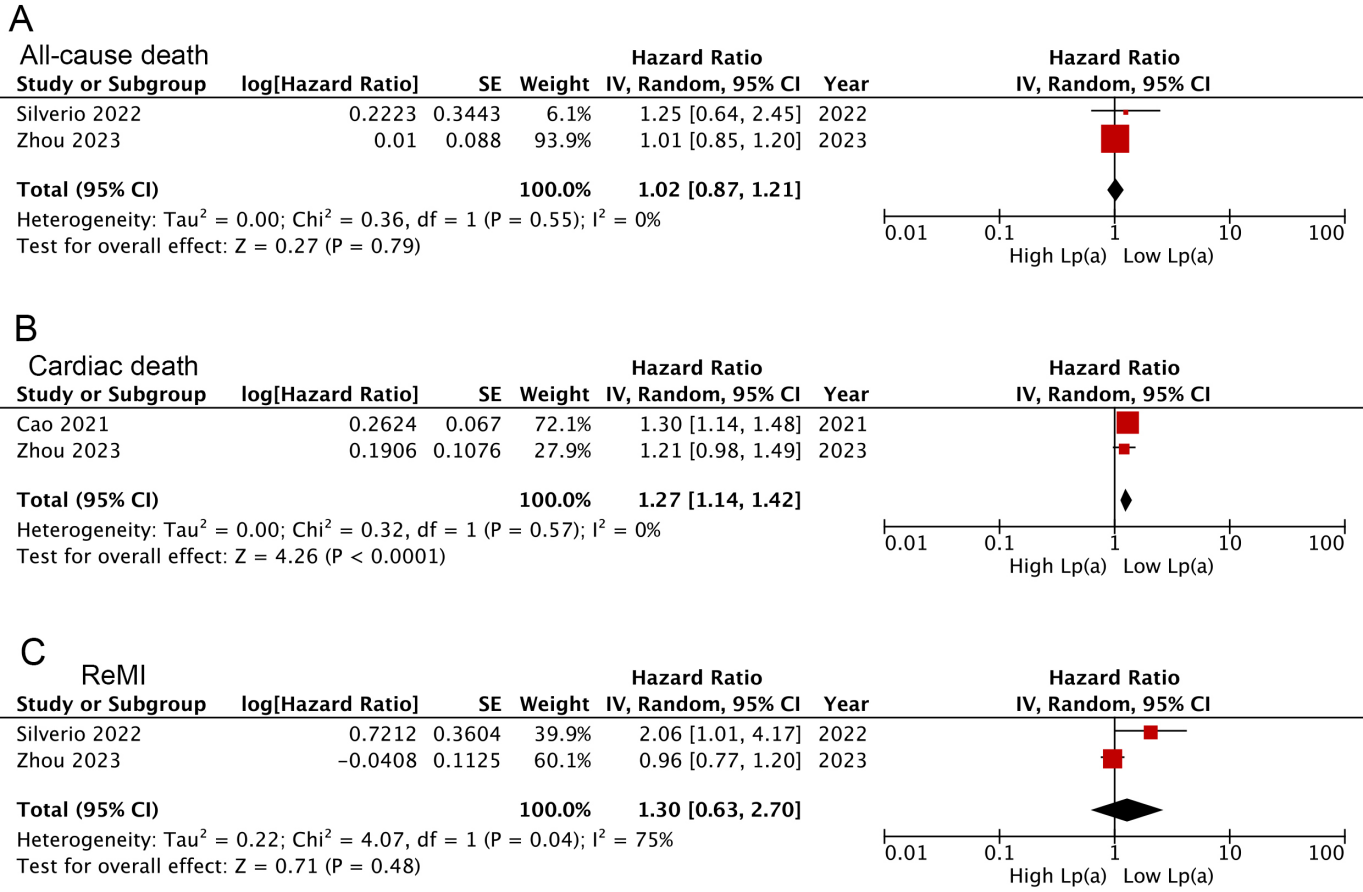
**Forest plots related to meta-analyses for the Lp(a) links following analysis as a continuous variable with the risk of all-cause death (A), cardiac death (B), and ReMI (C)**.

Overall, the combined findings of three studies [17, 25, 39] demonstrated that AMI patients categorized with the lowest serum Lp(a) tended to experience less cardiac death than those in the highest category (HR: 1.58, 95% CI: 1.07–2.34, I^2^ = 42%, *p *= 0.02) (Fig. 4B). Further, trends toward increased all-cause death [28, 30, 31, 33, 37, 39] (HR: 1.22, *p *= 0.20) and ReMI [21, 26, 33, 39] (HR: 1.20, *p *= 0.28) were also presented in those with the highest Lp(a), but not to levels of statistical significance (Fig. 4A,C). These results were consistent with findings when Lp(a) pooled as a continuous variable (cardiac death: HR: 1.27, 95% CI: 1.14–1.42, I^2^ = 0%, *p* < 0.0001; all-cause death: HR: 1.02, *p *= 0.79; ReMI: HR: 1.30, *p* = 0.48) (Fig. 5A–C).

### 3.5 Publication Bias

**Supplementary Fig. 4** presents funnel plots illustrating the relationship between Lp(a), calculated as a categorical or continuous variable, and MACEs. An apparent asymmetry was obtained, indicating a high risk of publication biases.

## 4. Discussion

In the current systematic review, we comprehensively analyzed the association between Lp(a) and the risk of MACEs after AMI. Our findings firstly indicated that higher Lp(a) levels correlated with an increased incidence of MACEs. Further, among subgroup analyses, significant associations between elevated Lp(a) and MACE prevalence among female individuals with DM or hypertension were observed; these links were not observed in the remaining subgroups. In addition, elevated Lp(a) was more seemingly related to long-term rather than short-term MACE occurrences. Altogether, these findings are likely more applicable to female populations with coincident DM or hypertension and more suitable to evaluate the prolonged prognosis following AMI.

No prior systematic reviews and meta-analyses have specifically addressed this topic solely relating to populations surviving AMI, despite substantial evidence illustrating the great harms of elevated Lp(a) in the secondary prevention of recurrent cardiovascular events. Willeit *et al*. [42] pooled seven randomized controlled trials with 29,069 patients in a review and highlighted those high levels of both baseline and on-statin Lp(a) exhibited an independent approximately linear relation with incident cardiovascular disease in the general population receiving lipid-lowering therapy. In addition, Wang *et al*. [43] performed a systematic review enrolling 17 studies that indicated a similar relationship between Lp(a) and cardiovascular risks for populations with established coronary artery disease. Notably, a large percentage of the population with dyslipidemia could not gain noticeable benefits from statins or proprotein convertase subtilisin/kexin type 9 (PCSK9) inhibitors in secondary prevention therapies [42]. However, these might involve one Lp(a)-associated mechanism that requires an absolute reduction in Lp(a) levels for a clinically apparent decrease in cardiac risk, as highlighted by the Mendelian randomization study [44, 45]. In contrast, routine lipid-lowering drugs could not sufficiently eliminate elevated Lp(a) levels. Moreover, a meta-analysis published in 2020 demonstrated that LDL-C content exhibited an apparent association with incident cardiovascular disease only when the Lp(a) cholesterol content was incorporated into its measurement [46]. Notably, the ODYSSEY OUTCOMES trial has recently demonstrated that alirocumab-related MACE reductions might be mediated via decreased Lp(a) levels [47]. These results indirectly indicate the significant increase in Lp(a)-related risk for recurrent MACEs, which aligns with our findings in the current comprehensive meta-analysis among patients surviving AMI.

Regarding the Lp(a) positive relationship with MACEs after AMI, we found that it might be primarily mediated by the increased cardiovascular mortality in relation to elevated Lp(a); these findings conformed to the Emerging Risk Factors Collaboration, which reported that an elevation in Lp(a) of 3.5-fold correlated with an approximately 14% increase in cardiovascular death; meanwhile, no apparent association was presented for the risk of non-vascular mortality [48]. Overall, these analyses strongly demonstrated that Lp(a)-mediated coronary damage is potentially mainly responsible for the worse prognosis after AMI. Some genetic and epidemiological studies have shown that elevated Lp(a) was associated with the prevalence and progression of myocardial infarction [49], atherosclerotic stenosis [50, 51], as well regarding aortic valve calcification [52, 53]; however, certain mechanisms related to these conditions may contribute to the link between higher Lp(a) levels and an augmented risk of cardiovascular events [47]. Briefly, Lp(a) has been implicated in promoting aortic valve sclerosis and calcification [12], which may contribute to developing aortic stenosis and increasing the burden on the cardiovascular system in AMI patients. Additionally, Lp(a) could aggravate left ventricular hypertrophy [13], a common complication of AMI, by promoting inflammation and fibrosis, which might worsen myocardial function and increase the risk of adverse outcomes. Possible pathophysiological mechanisms might explain these observed correlations. The lipotoxic composition of Lp(a) (low-density lipoprotein-like core, etc.) could be transmitted to the injured vessel walls, causing endothelial dysfunction, inflammation, and consequential atherosclerosis [54, 55]. However, some researchers have also elucidated the prothrombotic roles [56] and anti-fibrinolytic functions of Lp(a) [57]. Lp(a) might interfere with plasminogen activity owing to molecular similarity, leading to a deceleration in fibrinolysis and an indirect promotion of thrombosis. A recent study has shown that combining Lp(a) with fibrinogen and hs-CRP can significantly improve the accuracy of cardiovascular risk prediction [58].

Conversely, the connection between Lp(a) and MACEs seems to exist in females and individuals with DM or hypertension. In diabetic patients, the synergistic effect of high blood glucose and elevated Lp(a) may amplify pro-inflammatory and prothrombotic pathways. High blood glucose contributes to increased oxidative stress and endothelial dysfunction, which may enhance the proinflammatory properties of Lp(a) and promote thrombogenesis, further increasing the risk of adverse cardiovascular outcomes [59]. Similarly, high Lp(a) may worsen the effects of elevated blood pressure in hypertensive patients by increasing vascular stiffness and promoting plaque formation in the arteries, thereby exacerbating the progression of cardiovascular disease. In females, the potential hormonal influence on Lp(a) levels should be considered, as postmenopausal women tend to have higher Lp(a) levels, which may increase their risk of cardiovascular events compared to matched male counterparts [60]. These findings made it easy to understand why the effects of Lp(a) on incident MACEs varied in subgroup populations with or without certain risk factors. Notably, the varying prognostic significance of Lp(a) implied that Lp(a) might act differently in promoting cardiovascular events in populations with and without these above-mentioned risk indicators. Hence, heightened emphasis should be placed on Lp(a) within clinical practice owing to its intricate impact on cardiovascular disorders.

Our meta-analysis still possessed some limitations. Firstly, the included studies had diverse cut-off values of Lp(a), definitions of MACEs, and enrolled populations with varying characteristics, potentially leading to evident heterogeneity. Thus, the pooled findings regarding the relationship between Lp(a) and MACE incidents should be interpreted cautiously. Secondly, due to varied Lp(a) cut-off values among included studies, we could not establish a suitable threshold to distinguish elevated Lp(a). Thirdly, all included studies were cohorts, which limited the ability to establish a causal association between Lp(a) and the occurrence of MACEs. Lastly, factors such as variations in in-hospital management across institutions may have potential prognostic implications for populations surviving AMI, which could partly impact the significance of the conclusions drawn in this review.

## 5. Conclusions

In conclusion, Lp(a) was positively associated with MACE incidents, which might primarily be mediated by increased cardiovascular death. This finding seems more applicable to evaluating the long-term prognosis after AMI among female individuals with concomitant DM and/or hypertension. Hence, it is imperative to ascertain further whether Lp(a)- lowering treatment could reduce MACEs and ultimately improve the prognosis of patients surviving AMI. Meanwhile, a rationale for popularizing Lp(a) measurements in patients suffering from AMI should be provided.

## Availability of Data and Materials

All data generated can be obtained from this published article and its additional information files.

## Supplementary Material

Supplementary material associated with this article can be found, in the online version, at https://doi.org/10.31083/RCM27376.
