# Supplementary material for: The Association of Lipoprotein(a) with Major Adverse Cardiovascular Events after Acute Myocardial Infarction: A Meta-Analysis of Cohort Studies
Source: Rev Cardiovasc Med. 2025 May 15;26(5):27376. doi: 10.31083/RCM27376 (PMC12135674; doi:10.31083/RCM27376)
Supplement: Supplementary file 1 [file 2153-8174-26-5-27376-s1.zip › Supplementary Material.zip!/Supplementary materials/Supplementary Table 1.docx]

**Table S1**

**PubMed 2024.02.14 477**

**((((((((((((((((((((("Anterior Wall Myocardial Infarction"[Title/Abstract]) OR ("Inferior Wall Myocardial Infarction"[Title/Abstract])) OR (MINOCA[Title/Abstract])) OR ("Non-ST Elevated Myocardial Infarction"[Title/Abstract])) OR ("Shock, Cardiogenic"[Title/Abstract])) OR ("ST Elevation Myocardial Infarction"[Title/Abstract])) OR ("Infarction, Myocardial"[Title/Abstract])) OR ("Infarctions, Myocardial"[Title/Abstract])) OR ("Myocardial Infarctions"[Title/Abstract])) OR ("Cardiovascular Stroke"[Title/Abstract])) OR ("Cardiovascular Strokes"[Title/Abstract])) OR ("Stroke, Cardiovascular"[Title/Abstract])) OR ("Strokes, Cardiovascular"[Title/Abstract])) OR ("Myocardial Infarct"[Title/Abstract])) OR ("Infarct, Myocardial"[Title/Abstract])) OR ("Infarcts, Myocardial"[Title/Abstract])) OR ("Myocardial Infarcts"[Title/Abstract])) OR ("Heart Attack"[Title/Abstract])) OR ("Heart Attacks"[Title/Abstract])) OR ("Myocardial Infarction"[Mesh]))) AND (("Lipoprotein(a)"[Mesh]) OR ((((Lipoprotein Lp(a)[Title/Abstract])) OR (Lipoprotein (a)[Title/Abstract]))) OR (Lipoprotein a[Title/Abstract])) OR (Lp(a)[Title/Abstract]))))**

**Embase 349 2024.02.14**

**('Lipoprotein(a)'/exp OR 'Lipoprotein Lp(a)':ab OR 'Lipoprotein (a)':ab OR 'Lipoprotein a':ab OR 'Lp(a)':ab) AND ('acute heart infarction'/exp OR 'infarction, myocardial':ab OR 'infarctions, myocardial':ab OR 'acute myocardial infarctions':ab OR 'acute cardiovascular stroke':ab OR 'acute cardiovascular strokes':ab OR 'stroke, cardiovascular':ab OR 'strokes, cardiovascular':ab OR 'acute myocardial infarct':ab OR 'infarct, myocardial':ab OR 'infarcts, myocardial':ab OR 'acute myocardial infarcts':ab OR 'acute heart attack':ab OR 'acute heart attacks':ab OR 'anterior wall myocardial infarction':ab OR 'inferior wall myocardial infarction':ab OR 'minoca':ab OR 'non-st elevated myocardial infarction':ab OR 'shock, cardiogenic':ab OR 'st elevation myocardial infarction':ab)**

**Web of science 992 2024.02.14**

\# 1 (TS=(**“Lipoprotein(a)”**) OR (AB=(**“Lipoprotein Lp(a)” OR “Lipoprotein (a)” OR “Lipoprotein a” OR “Lp(a)”**))

**#2 (AB=(“acute heart infarction” OR “infarction, myocardial” OR “infarctions, myocardial” OR “acute myocardial infarctions” OR “acute cardiovascular stroke” OR “acute cardiovascular strokes” OR “stroke, cardiovascular” OR “strokes, cardiovascular” OR “acute myocardial infarct” OR “infarct, myocardial” OR “infarcts, myocardial” OR “acute myocardial infarcts” OR “acute heart attack” OR “acute heart attacks” OR “anterior wall myocardial infarction” OR “inferior wall myocardial infarction” OR “minoca” OR “non-st elevated myocardial infarction” OR “shock, cardiogenic” OR “st elevation myocardial infarction”)) OR (TS=(“acute heart infarction”))**

**#1 AND #2**

**MEDLINE 188 2024.02.14**

**MESH subheading, Title, Abstract**

**('Lipoprotein(a)' OR 'Lipoprotein Lp(a)' OR 'Lipoprotein (a)' OR 'Lipoprotein a' OR 'Lp(a)')**

**'acute heart infarction' OR 'infarction, myocardial' OR 'infarctions, myocardial' OR 'acute myocardial infarctions' OR 'acute cardiovascular stroke' OR 'acute cardiovascular strokes' OR 'stroke, cardiovascular' OR 'strokes, cardiovascular' OR 'acute myocardial infarct' OR 'infarct, myocardial' OR 'infarcts, myocardial' OR 'acute myocardial infarcts' OR 'acute heart attack' OR 'acute heart attacks' OR 'anterior wall myocardial infarction' OR 'inferior wall myocardial infarction' OR 'minoca' OR 'non-st elevated myocardial infarction' OR 'shock, cardiogenic' OR 'st elevation myocardial infarction' OR 'acute myocardial infarction'**

**Cochrane library 23 2024.02.14**

**ID	Search	Hits**

**#1	"Lipoprotein(a)"	1682**

**#2	"Lipoprotein Lp(a)"	48**

**#3	“Lipoprotein (a)”	1682**

**#4	“Lipoprotein a”	1682**

**#5	“Lp(a)”	1153**

**#6	#1 OR #2 OR #3 OR #4 OR #5	2100**

**#7	“acute heart infarction”	3256**

**#8	“infarction, myocardial”	2754**

**#9	“infarctions, myocardial”	0**

**#10	“acute myocardial infarctions”	78**

**#11	“acute cardiovascular stroke”	0**

**#12	“acute cardiovascular strokes”	0**

**#13	“stroke, cardiovascular”	186**

**#14	“strokes, cardiovascular”	16**

**#15	“acute myocardial infarct”	133**

**#16	“infarct, myocardial”	6**

**#17	“infarcts, myocardial”	0**

**#18	“acute myocardial infarcts”	5**

**#19	“acute heart attack”	28**

**#20	“acute heart attacks”	7**

**#21	“anterior wall myocardial infarction”	137**

**#22	“inferior wall myocardial infarction”	20**

**#23	“minoca”	40**

**#24	"non-ST elevated myocardial infarction"	151**

**#25	“shock, cardiogenic”	435**

**#26	“st elevation myocardial infarction”	2959**

**#27	#7 OR #8 OR #9 OR #10 OR #11 OR #12 OR #13 OR #14 OR #15 OR #16 OR #17 OR #18 OR #19 OR #20 OR #21 OR #22 OR #23 OR #25 OR #26 OR #24	8961**

**#28	#27 AND #6	23**
